# 408. Safety and Immunogenicity of Meningococcal Serogroup B Vaccine (4CMenB) and 13-Valent Pneumococcal Vaccine (PCV13) Administered Concomitantly with Routine Infant Vaccines (RIVs) to Healthy United States (US) Infants: A Randomized Controlled Study

**DOI:** 10.1093/ofid/ofaf695.015

**Published:** 2026-01-11

**Authors:** Debasish Saha, Laura Tomasi Cont, Walter J Rok, Judith M Martin, Pavitra Keshavan, Danielle Morelle, Mauro Trapani, Elisa Cinconze, Maria Lattanzi, Daniela Toneatto

**Affiliations:** GSK Vaccines, Wavre, Brabant Wallon, Belgium; GSK, Siena, Toscana, Italy; Pediatric Associates of Fall River, Fall River, Massachusetts; University of Pittsburgh, Pittsburgh, PA; GSK, Siena, Toscana, Italy; GSK, Siena, Toscana, Italy; GSK, Siena, Toscana, Italy; GSK, Siena, Toscana, Italy; GSK, Siena, Toscana, Italy; GSK, Siena, Toscana, Italy

## Abstract

**Background:**

4CMenB (MenB-4C, GSK) has been licensed in the US since 2015 for individuals aged 10–25 years. This study assessed its safety and immunogenicity in US infants when administered concomitantly with PCV13 (Pfizer) and other US-recommended RIVs.

**Figure:**
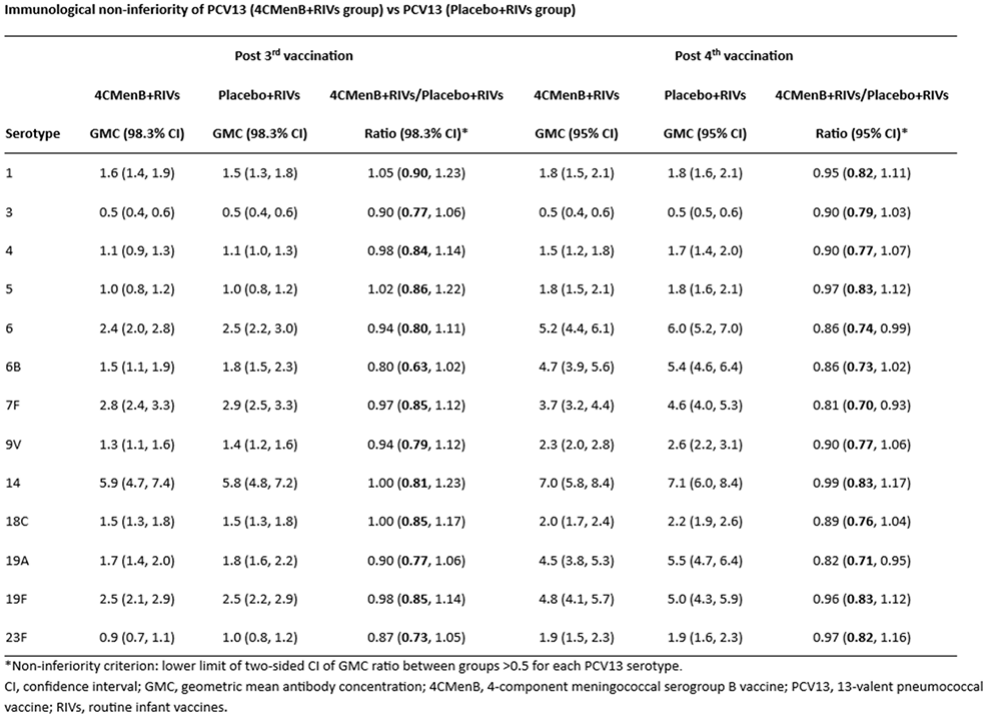

**Methods:**

This phase 3B, observer-blind, randomized, placebo-controlled, multicenter study (NCT03621670) randomized (2:1 ratio) 1195 healthy US infants aged 6–12 weeks to receive, at approx. 2, 4, 6, and 12 months of age, either 4CMenB administered concomitantly with PCV13 and other RIVs (N=786; 4CMenB+RIVs) or placebo with PCV13 and other RIVs (N=409; Placebo+RIVs). Primary objectives were to assess vaccine safety, demonstrate sufficiency of immune responses to 4CMenB by human serum bactericidal assay (hSBA) against each of 4 serogroup B (MenB) indicator strains and all strains combined (composite), and non-inferiority of pneumococcal serotype-specific geometric mean antibody concentrations (GMCs).

**Results:**

Pre-specified sufficiency criteria 1 month post-dose 3 (based on lower limit [LL] of 99.2% confidence interval [CI] for hSBA titers ≥lower limit of quantitation [LLOQ]) and post-dose 4 (based on LL of 95.8% CI for hSBA titers ≥8/16) were met for fHbp, NadA, and PorA strains, but not for NHBA and the composite response. For NHBA, the CI LL for percentage of participants with hSBA titers ≥8 post-dose 4 was 72.5% (sufficiency criterion: ≥75%). Percentages of participants with hSBA titers ≥LLOQ against each MenB indicator strain post-dose 4 were 86.1%–99.6% (75.7%, composite) in the 4CMenB+RIVs group vs 0.0%–2.8% (0.0%, composite) in the Placebo+RIVs group. The success criterion for immunological non-inferiority of PCV13 (4CMenB+RIVs) vs PCV13 (Placebo+RIVs) was met for each PCV13 antigen (see table). 4CMenB was well tolerated, with no major between-group differences in unsolicited adverse events and no safety concerns identified.

**Conclusion:**

4CMenB was well tolerated and immunogenic in US infants, with no clinically significant interference against concomitantly administered RIV. Comparison against the 4CMenB-naïve group shows MenB immune responses in the 4CMenB+RIVs group were elicited solely by 4CMenB.

Acknowledgements: Enovalife Medical Communication Service Center, on behalf of GSK (writer: J. Knowles).

Funding: GSK.

**Disclosures:**

All Authors: No reported disclosures

